# Sex differences in the trajectories of and factors related to extracurricular sport participation and exercise: a cohort study spanning 13 years

**DOI:** 10.1186/s12889-020-09745-8

**Published:** 2020-11-02

**Authors:** Wen-Chi Wu, Ling-Yin Chang, Dih-Ling Luh, Chi-Chen Wu, Fiona Stanaway, Lee-Lan Yen, Hsing-Yi Chang

**Affiliations:** 1 Department of Health Promotion and Health Education, National Taiwan Normal University, Taipei, Taiwan; 2 Institute of Health Behaviors and Community Sciences, School of Public Health, National Taiwan University, No.17, Xuzhou Rd., Zhongzheng Dist, Taipei City, 100 Taiwan; 3 Department of Public Health, Chung Shang Medical University, Taichung, Taiwan; 4 Institute of Population Health Sciences, National Health Research Institutes, No.35, Keyan Road, Zhunan, Miaoli County 350 Taiwan; 5 School of Public Health, University of Sydney, Sydney, Australia; 6 Institute of Health Policy and Management, School of Public Health, National Taiwan University, Taipei, Taiwan

**Keywords:** Extracurricular exercise, Repeated-measures latent class analysis, Childhood, Adolescence, Emerging adulthood

## Abstract

**Background:**

Extracurricular sport participation and exercise (ESPE) refers to regular exercise/sport participation in addition to the physical education in school among a school-aged population. Rather than general physical activity, ESPE is typically deliberately initiated and presents an efficient target for interventions. However, compared to physical activity, relatively few studies have investigated sex differences in the development of and factors associated with ESPE using a person-centered approach. This study aimed to examine the latent trajectories of ESPE from childhood to emerging adulthood across sexes, and to identify the associated sex-specific individual (i.e., body mass index, body dissatisfaction, stress, and screen behavior) and parental (i.e., parental exercise and parental screen behavior) factors.

**Methods:**

This study used data from part of the Child and Adolescent Behavior in Long-term Evolution (CABLE) project, which comprised 2072 fourth graders (aged 9 years) in Northern Taiwan followed annually from 2001 to 2013 (13 waves). Repeated-measures latent class analysis was used to identify the trajectories of ESPE for males and females, respectively. Multinomial logistic regression was further used to identify sex-specific factors related to ESPE.

**Results:**

Four trajectories of ESPE were identified for males and females. For males, these trajectories were Rarely-to-Never (20%), Often-to-Rarely (32%), Always-to-Never (21%), and Always (27%). For females, these trajectories were Rarely-to-Never (34%), Rarely (23%), Always-to-Rarely (33%), and Always (10%). We observed that the developmental patterns of ESPE varied by sex such that there was an earlier decline in the trajectories of ESPE in females than in males and that, compared with males, fewer females maintained exercise habits in young adulthood. Furthermore, we found several sex-specific factors related to ESPE, namely, stress, BMI, and parental exercise. Body dissatisfaction and individual screen behavior were associated with trajectories of ESPE for both sexes.

**Conclusions:**

We found distinct trajectories of ESPE from childhood to emerging adulthood for both sexes. The trajectories of ESPE for males and females, however, differ in terms of patterns and associated factors. Our findings suggest that efforts to increase ESPE should be initiated early, and may be made more effective by considering sex differences.

**Supplementary Information:**

The online version contains supplementary material available at 10.1186/s12889-020-09745-8.

## Background

The World Health Organization (WHO) identifies physical inactivity as the fourth leading cause of global mortality [1]. Enhancing physical activity can yield substantial benefits for physical health, mental health, academic achievement, and cognitive outcomes [2–4]. Despite recognition that engagement in regular physical activity should be developed early in life, namely during childhood and adolescence, 81% of adolescents across the world do not meet WHO recommendations regarding physical activity [5, 6]. Instead of targeting physical activity, exercise and sport may be the more efficient areas in which to intervene [7, 8]. Exercise is a subcategory of physical activity that is planned, structured, repetitive and purposeful bodily movement in the sense that the improvement or maintenance of one or more components of physical fitness is the objective [8–10]. Sports includes all forms of competitive physical activities which are governed by formal or informal rules [8, 11]. In general, sport participation and exercise are the major sources of activities that are of sufficient intensity and duration to yield moderate-to-vigorous energy expenditure for most of the populace [7, 8]. Thus, increasing the quantity of sport participation and exercise will inevitably escalate the overall level of physical activity.

Extracurricular sport participation and exercise (ESPE), referring to regular exercise/sport participation in addition to the normal physical education, is a significant form of health-promoting physical activity [8, 12]. Normal physical education is typically disciplined and ruled by school and government policies. For instance, in Taiwan, every school is required to provide at least 2 h of physical education for each student per week. However, given the tight school schedule, there is typically no other free time in which to exercise. Most students participate in physical education to fulfill school requirements. However, these requirements may not be sufficient to meet the recommendation of at least 60 min of moderate-to-vigorous physical activity (MVPA) each day [4]. According to the information from the 2001 National Health Interview Survey in Taiwan, only 28.4% of adolescents and 21% of adults met recommended guidelines [13, 14]. Thus, increasing the level of ESPE is a means to compensate for this deficit.

Although numerous longitudinal studies have identified a declining pattern of exercise/sport and physical activity during adolescence [15–19], not everyone has reduced the frequency with which they exercise. When the developmental patterns were retrieved using a person-centered approach, such as group-based trajectory modeling and repeated-measures latent class analysis, some studies found that a group of participants either maintained a high level of activity or delayed the decline in activity [19]. For example, a longitudinal study found that a group of adolescents (21%) maintained a stable higher level of MVPA from age 14 to 18 [20]. Similarly, Kwon and his colleagues identified a group of children (18%) who consistently maintained MVPA from age 5 to 19, and they further found that almost half of the children (46%) participated in organized sports during the follow-up time [21]. In addition, a recent reviewed article found that three out of 11 studies that retrieved trajectories of physical activity or sport participation among young populations discovered a highly active pattern of physcial activity [19]. Based on previous findings, it is likely that adolescents may follow different developmental patterns of extacurricular exercise/sport, although no studies have specifically focused on this issue.

Sex differences in exercise/sport and physical activity have been well-established by scholars [22]. However, mixed findings in the existing literature suggest that the role of sex in the development of exercise/sport still requires further investigation. In terms of the longitudinal patterns of physical activity, Farooq et al. [17] found that while a subgroup of boys (18.8%) maintained a high level of MVPA from the age of 7 to 15 years, the level of MVPA for the remaining boys and all the girls decreased. Another study found that one third of both the boys and girls continued high-frequency organized physical activity from age 4 to 17 years [23]. There is also a study which found that only 9% of the girls but a third of the boys maintained MVPA [24]. Addtionally, regarding the rates of change in vigorous activity, a longitudinal study monitoring adolescents from the age of 7 to 11 years found that physical activity among girls decreased more rapidly than among boys [25], while another study found no significant difference in rates of decline between sexes [17]. Moreover, pertaining to the context of physical activity, a longitudinal study following a group of American adolescents from the age of 10 to 12 years found that girls increased MVPA during after-school hours, whereas boys maintained MVPA; however, no sex difference was found in school-time MVPA [18]. These inconsistent results substantiate the need to understand the effects of sex differences in the distinct developmental patterns of ESPE and factors that contribute to such disparities.

Regarding factors associated with ESPE, reseach [26–29] has found that children and adolescents’ exercise/sport participation could be influenced by factors of different contexts according to Bandura’s Social Learning/Cognitive Theory [30] and Bronfenbrenner’s bioecological model [31]. Among many contexts, family is the most important in terms of helping children to develop exercise habits [26]. Specifically, parental modeling and support affect children’s physical activity by the observational learning process and positive outcome expectations [32]. For example, one study proved that parental organized physical activity increased their children’s extracurricular sport participation [33]. Another study showed that parental sedentary behavior was also associated with children’s physical inactivity [34]. In addition, both theories emphasize the contributions of individual factors on the development of children’s behavior. There is also empirical evidence of several individual characteristics that influence sport participation and exercise among children, including physical factors, such as body mass index (BMI) [35], psychological factors, such as body dissatisfaction [36] and stress [37], as well as behavior factors, such as sedentary behavior [38].

There is also evidence of sex differences in the important individual and parental determinants of exercise/sport participation. Regarding individual factors, females with a lower BMI were found to have a higher probability of exercising, whereas body mass had no significant association with exercise in males [39]. Additionally, higher body dissatisfaction (indicated by greater body shame and higher appearance anxiety) was associated with decreased rates of participation in physical activities by females [36] but increased exercise engagement among males [40]. Furthermore, males with a higher level of stress might be more likely to exercise than females because males are inclined to use exercise as a coping strategy [41]. Moreover, males who reported more on-screen time were more likely to fail to achieve the recommended MVPA level due to lower availability of time for exercise, but this association was not found for females [42]. Regarding parental factors, parental exercise was found to only associate with boys’ exercise participation [43, 44]. Research has also demonstrated sex differences in influences of parents’ screen behavior on their offspring’s screen behavior [45], which could result in different levels of sport participation and exercise in boys and girls. However, whether these factors have sex-specific associations with trajectories of ESPE is yet to be explored.

**Fig. 1:**
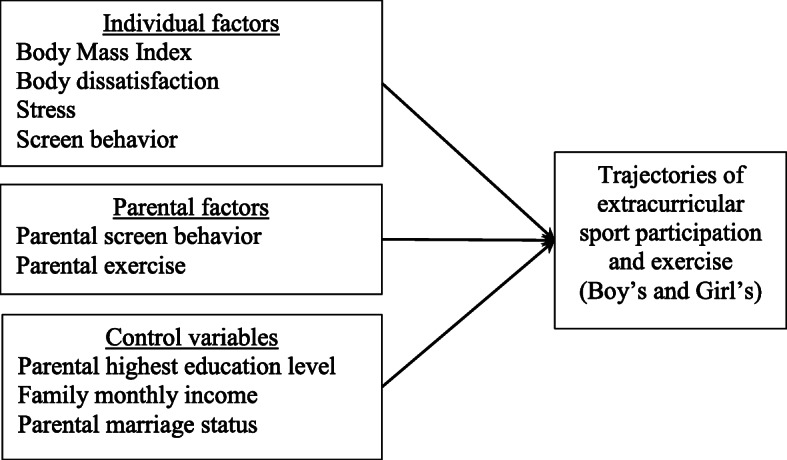
Study framework

To fill these gaps in the literature, this study aimed to (1) depict the trajectories of ESPE in males and females from childhood to emerging adulthood, and (2) identify sex differences in factors associated with ESPE trajectories. Figure 1 shows the study framework; it indicates the associations of individual and parental factors as well as the control variables with sex-specific ESPE trajectories. To the best of our knowledge, this is the first longitudinal study focusing on the sex-specific developmental patterns of ESPE over a long lifespan (i.e., 13 time points) and further examining whether factors related to these distinct patterns vary by sex. Establishing the developmental patterns of ESPE, such as whether they are increasing, decreasing, or being maintained, and their associated factors can help facilitate the development of effective exercise-enhancing programs.

## Methods

### Participants, design and setting

Data for this secondary analysis were obtained from the Child and Adolescent Behaviors in Long-term Evolution (CABLE) project. The study commenced in 2001 and followed participants annually until 2016. The CABLE project aimed to investigate the development of healthy behaviors from childhood to adolescence based on a socioecological model. The project incorporated multi-level factors including individual, interpersonal, organizational, and community regarding health lifestyle and health behavior. For example, individual-level factors included sociodemographic characteristics, personality, and lifestyle of students. Interpersonal-level factors consisted of family interaction, family support, family conflict, and peer relationship. In terms of organizational-level factors, the CABLE project collected information regarding several school characteristics such as size and facilities. Finally, the community-level factors included community cohesion and safety.

Participants were cluster-sampled from all public elementary schools in Taipei City and Hsinchu County in Taiwan based on a list of names provided by the Ministry of Education in 2001. Nine schools from each area were selected. Two cohorts, first and fourth graders in each school, were followed. Further details regarding the sampling process, sample size calculations, and instrument development are described elsewhere [46]. Signed informed consent was provided by the parents or primary caregivers of all participating students.

In the CABLE project, data were collected from students and their parents. Regarding the students, first to ninth graders completed their questionnaires in the classroom under the supervision of trained instructors. From the 10th grade onwards, students were interviewed individually by trained interviewers. The questionnaire consisted of two sections, namely self-administered, including private questions such as health behaviors and emotions, as well as interviewer filled-in, including sociodemographic background and school-related questions. The interviewers may interview the participants in any place they felt comfortable, such as a fast-food store or the student’s home, and the completed questionnaire was mailed to the CABLE team. The measurement of ESPE was included in the self-administered section. In the first four annual assessments, students also brought the maternal- and paternal-versions of the questionnaires home to their parents or primary caregivers. The questionnaires, which inquired about the parental education level, family income, marital status, and parental behaviors, were completed by the primary caregivers and then mailed back to the research team using a sealed envelope. The project was approved by the Human Research Medical Ethics Committee of the National Health Research Institutes in Taiwan (EC9009003).

In this study, we analyzed data from the second cohort (fourth graders), who were followed and interviewed annually from 2001 to 2013 (aged 9 to 21 years, 13 waves). The completion rate for each year was equal to the number of participants who completed the questionnaire in a specific year divided by the number of participants in the first year. During the study period, the completion rate ranged from 81.6 to 98.1%. The final analytical sample comprised 2072 participants (1075 males and 997 females) who were enrolled in 2001 and who provided at least one wave of data on the measure of ESPE. Overall, 46.53% of participants provided ESPE data in all 13 waves, and 13.61, 7.53, 7.92, 6.03, 4.01, 2.12, 3.47, 1.69, 1.30, 1.88, 0.97, and 2.94% of participants provided ESPE data in 12 waves to one wave, respectively. Reasons for the attrition rate included moving, refusal to be interviewed, health issues, and loss of contact.

### Measures

#### ESPE

The measurement of ESPE was assessed annually by asking participants “Not counting normal physical exercise courses in school, have you exercised/participated in sports in the past week?”. The possible responses were “1 = never,” “2 = rarely (one or two days),” “3 = often (three to six days),” and “4 = always (every day).” The inter-method reliability was used to examine the reliability of this one-item measurement by correlating it with another three-item measure of physical activity assessed in the 12th wave that determined whether individuals met the WHO’s recommendation of physical activity. A positive association was found between the one-item and three-item measurements, and indicated good reliability of the one-item measurement used in the current study. Appendix 1 provides the detailed information of the calculation.

#### Related factors

This study examined the sex-specific effects of several individuals and parental factors on the trajectories of ESPE. Specific measures for each factor are described as follows.

##### Individual factors

All individual factors were measured from 2001 (aged 9 years) to 2006 (aged 14 years), and scores at each wave were averaged to reflect the mean levels of each factor. BMI was measured by self-reported weight (kg) divided by the square of height (m^2^). The current BMI measure had high correlations with the objective measures of BMI deriving from the height and weight assessed by school nurses in the 2nd and 6th waves (*r* = 0.85 and 0.84, respectively). The good agreement between methods indicates that the self-reported height and weight is a reliable proxy measure of BMI [47]. *Body dissatisfaction* was measured using four items, namely self-perceived satisfaction with appearance, figure, height, and weight [48], all of which were rated on a 5-point scale ranging from 1 (very satisfied) to 5 (very unsatisfied). The body dissatisfaction score was obtained by summing the four items, with higher scores indicating a higher level of body dissatisfaction (Cronbach’s alpha of 0.68 to 0.72). *Stress* was assessed using questions adapted from a previous study [49] that asked participants to rate their perceived levels of stress from eight different sources (e.g., academic performance, relationships with friends, and relationships with parents). All items were rated on a 5-point scale ranging from 0 (absolutely no stress) to 4 (extremely high-level stress) and were summed to create an overall stress score (Cronbach’s alpha of 0.79 to 0.84). *Screen behavior* was assessed using two items: “Have you used a computer or played video games continuously for more than two hours in the past week?” and “Have you watched television continuously for more than two hours in the past week?” All responses were rated on a four-point scale ranging from 1 (never) to 4 (every day). The level of screen behavior was computed by summing the frequencies of these two items. Because screen behavior was measured by two-items, the Spearman-Brown coefficient was the most appropriate reliability statistic [50]. The Spearman-Brown coefficients for screen behavior were from 0.52 to 0.69 across the six waves.

##### Parental factors

*Parental* exercise was measured by one item adapted from other survey studies [51, 52] asking parents “Have you exercised in the past week?” and was dichotomized as “regular exercise” (defined as at least one parent had often or always exercised) and “irregular exercise or no exercise.” *Parental screen behavior* was measured at each wave from 2001 to 2004 using the same two items that assessed students’ screen behavior. Response categories for these two items, which ranged from 1 (never) to 5 (always), were summed and averaged across 4 years to reflect the level of parental screen behavior. Spearman-Brown coefficients of parental screen behavior ranged from 0.28 to 0.34, which falls in the optimal range (i.e.,0.2–0.4) according to Briggs and Cheek’s suggestion [53].

#### Control variables

Parental education, household income, and marital status were included as control variables based on the previous evidence of associations between these social demographic variables and exercise [39]. All control variables were collected between 2001 and 2004 and reported by parents or primary caregivers. *Parental education* was measured as the highest level of education attained by either parent and was coded as low (less than or equal to 12 years) or high (more than or equal to 13 years). *Monthly household income* was averaged across 4 years and categorized as low (less than 59,999 new Taiwan dollars (NTD; 1 NTD ≈ 0·03 $US), medium (60,000–119,999 NTD), or high (more than 120,000 NTD) income groups. *Parental marital status* was dichotomized as married or not married.

### Analytic procedure

Descriptive statistics included means, standard deviations, and study variable distribution. Student’s t-test and chi-square test were used to identify associations between the study variables and sex. Repeated-measures latent class analysis (RMLCA) was used to identify distinct ESPE trajectories from childhood to young adulthood [54]. RMLCA is a statistical method that can be used to cluster individuals into a number of latent classes based on the pattern of responses to the ESPE questions at discrete time points [54]. All models were estimated using SAS version 9.4 (SAS Institute Inc., Cary, NC) via Proc LCA. The number of latent patterns was determined using 1) fit indices, namely the Akaike Information Criterion (AIC), Bayesian Information Criterion (BIC), and sample size-adjusted BIC, where a lower value indicates a better model; 2) average classification probability (ACP), where a higher value indicates better classification; and 3) interpretation of latent groups. According to a recent simulation study, BIC was preferred when comparing models [55]. We performed multiple group comparisons to determine potential sex differences [56] and found that the latent classes of exercise varied by sex. RMLCA and subsequent analyses were therefore stratified by sex. Finally, multinomial logit models were used to examine associations between related factors and different trajectories of ESPE across sex.

### Missing data

In RMLCA, missing data were addressed using the maximum likelihood estimation [54]. For the multinomial logistic model, only complete data were used (*n* = 1701, 82.09%). We compared the analytic sample with those that had missing data and found no differences between the two samples in terms of sex, body dissatisfaction, stress, screen behavior, parental education, parental exercise, and parental screen behavior. However, those with missing data were significantly more likely to have a lower level of BMI (18.9 vs. 19.6), parents who were not married (18.6% vs. 12.8%), or parents with low education (32.7% vs. 29.16%). A sensitivity analysis was performed to assess the robustness of the findings. This involved comparing results by using a multiple imputation strategy with results applying a list-wise deletion technique [57]. Twenty sets of missing values were imputed when conducting multiple imputations by using Markov Chain Monte Carlo methods, and the results of each data set were then combined to perform multinomial logistic regressions.

## Results

### Sample characteristics

The descriptive statistics are presented in Table 1. Sex differences were evident in individual factors. For instance, males had higher BMI and engaged in more frequent screen behavior than females. By contrast, females were more dissatisfied with their body and expressed a higher level of stress than males. Among parental factors, no sex differences were identified. Most of the parents reported having exercised irregularly or not exercised in the past week (*n* = 1195, 62.57%). The average frequency of parental screen behavior ranged from rarely to sometimes. In terms of control variables, a greater proportion of males had parents with high-level education (41.46%) than did females (36.03%). There were no significant differences in other control variables across sex. The majority of parents were married (*n* = 1716, 86.14%) and had a monthly household income of between 60,000 and 120,000 NTD (equals to 2000 to 4000 USD) (*n* = 983, 49.62%).

**Table 1:** Descriptive statistics for multi-faceted related factors across sex

Related Factors	Total (*n* = 2072)			Male (*n* = 1075)			Female (*n* = 997)			*P*-value
	n	mean or %	SE	n	mean or %	SE	n	mean or %	SE	
*Individual factors*										
Body Mass Index (averaged)	1952	19.56	0.08	1013	20.04	0.12	939	19.03	0.10	< 0.001
Body dissatisfaction (Range: 4–20, averaged)	1791	11.92	0.07	924	11.15	0.10	867	12.74	0.10	< 0.001
Stress (Range: 0–32, averaged)	1792	10.10	0.12	924	9.24	0.17	868	11.01	0.17	< 0.001
Screen behavior (Range: 2–8, averaged)	2011	3.91	0.03	1043	4.19	0.04	968	3.60	0.03	< 0.001
*Parental Factors*										
Parental screen behavior (Range: 2–10, averaged)	1995	4.84	0.03	1035	4.87	0.04	960	4.79	0.04	0.165
Parental exercise										
Regular	715	37.43%		366	37.12%		349	37.77%		0.769
Irregular or no exercise	1195	62.57%		620	62.88%		575	62.23%		
*Control variables*										
Parental highest education level										
Low (<= 12 years)	1256	61.15%		624	58.54%		632	63.97%		0.012
High (> = 13 years)	798	38.85%		442	41.46%		356	36.03%		
Family monthly income (averaged)										
Low	584	29.48%		287	27.92%		297	31.16%		0.083
Medium	983	49.62%		508	49.42%		475	49.84%		
High	414	20.90%		233	22.67%		181	18.99%		
Parental marriage status										
Not married	276	13.86%		147	14.22%		129	13.47%		0.628
Married or living together	1716	86.14%		887	85.78%		829	86.53%		

Individual-level factors were averaged based on available data from 2001 to 2006

Parental level factors and control variables were averaged based on available data from 2001 to 2004

### Trajectories of ESPE from ages of 9 to 21 years

Table 2 displays the results of fit indices for LCA models with different numbers of latent groups. For males, the log-likelihood statistic fell substantially when the number of latent classes increased to four. The four-class model also had the lowest BIC (14,627.51) and high ACP (0.83), indicating that it was the best-fitting model. For females, the model with four groups was also the best-fitting, with the lowest value of BIC (12,953.44) and a relatively high ACP (0.84).

**Table 2:** Determining the number of latent groups

Number of latent classes	Log-likelihood	AIC	BIC	Adjusted BIC	ACP
Males (*n* = 1075)					
1	−14,883.36	15,712.29	15,906.51	15,782.64	1.00
2	−14,170.26	14,366.09	14,759.51	14,508.59	0.92
3	−13,968.68	14,042.93	14,635.56	14,257.59	0.86
**4**	**−13,825.06**	**13,835.68**	**14,627.51**	**14,122.50**	**0.83**
5	−13,718.09	13,701.75	14,692.79	14,060.72	0.83
6	−13,619.73	13,585.02	14,775.26	14,016.14	0.81
Females (*n* = 997)					
1	−13,383.32	13,826.91	14,018.19	13,894.33	1.00
2	−12,776.92	12,694.10	13,081.58	12,830.67	0.91
3	−12,586.32	12,392.91	12,976.58	12,598.63	0.85
**4**	**−12,436.66**	**12,173.58**	**12,953.44**	**12,448.44**	**0.84**
5	−12,337.51	12,055.29	13,031.33	12,399.30	0.82
6	−12,257.19	11,974.65	13,146.89	12,387.81	0.82

*AIC* Akaike Information Criterion, *BIC* the Bayesian Information Criterion; Adjusted BIC, sample size-adjusted BIC. *ACP* average classification probability

Bolded letters indicate selected models

Table 3 shows the item-response probabilities for males. Class 1 was the largest class (32% of males) and was labeled as “Often-to-Rarely.” Children who belonged to the Often-to-Rarely class had a high probability of often engaging in ESPE from the age of 9 years, but this changed to rarely engaging in ESPE after they entered high school (aged 14 years). Class 2 was labeled “Rarely-to-Never” (20%) and consisted of participants who rarely engaged in ESPE from the age of 9 years and then never engaged in ESPE when they were 17 years old. Class 3 accounted for 21% of males and was labeled as “Always-to-Never.” This included participants with a high probability of always engaging in ESPE from the age of 9 to 14 years and a high probability of never engaging in ESPE after the age of 18 years. Class 4 was labeled as “Always” (27%) and consisted of participants with the highest probability of always engaging in ESPE over time. The average probability of correctly classifying participants into each latent class was 0.79, 0.86, 0.81, and 0.83 for classes 1, 2, 3, and 4, respectively.

**Table 3:** Item-response probabilities for a four-class model of extracurricular sport participation and exercise (ESPE) from ages 9 through 21 by sex

Latent class and membership probability	Male: Class 1				Male: Class 2				Male: Class 3				Male: Class 4			
	Often-to-Rarely				Rarely-to-Never				Always-to-Never				Always			
	32%				20%				21%				27%			
Age	Never	Rarely	Often	Always	Never	Rarely	Often	Always	Never	Rarely	Often	Always	Never	Rarely	Often	Always
9	0.06	0.32	**0.35**	0.28	0.14	**0.51**	0.22	0.14	0.09	0.19	0.27	**0.44**	0.04	0.11	0.24	**0.61**
10	0.03	0.27	**0.35**	0.36	0.12	**0.45**	0.27	0.16	0.00	0.14	0.31	**0.55**	0.01	0.04	0.16	**0.78**
11	0.00	0.21	**0.46**	0.34	0.16	**0.45**	0.28	0.11	0.03	0.07	0.29	**0.61**	0.01	0.03	0.08	**0.87**
12	0.02	0.27	**0.41**	0.30	0.27	**0.57**	0.12	0.04	0.02	0.17	0.33	**0.48**	0.02	0.04	0.19	**0.74**
13	0.04	0.28	**0.44**	0.25	0.21	**0.59**	0.15	0.05	0.04	0.20	0.28	**0.48**	0.01	0.04	0.24	**0.71**
14	0.02	**0.34**	**0.34**	0.30	0.29	**0.47**	0.16	0.08	0.09	0.24	0.26	**0.41**	0.00	0.08	0.17	**0.75**
15	0.08	**0.41**	0.35	0.15	0.44	**0.41**	0.07	0.08	0.22	**0.38**	0.19	0.20	0.08	0.14	0.23	**0.55**
16	0.02	**0.43**	0.41	0.15	0.37	**0.48**	0.08	0.06	0.34	**0.37**	0.17	0.12	0.03	0.09	0.25	**0.63**
17	0.07	**0.48**	0.29	0.16	**0.45**	0.38	0.06	0.12	0.36	**0.40**	0.14	0.10	0.05	0.15	0.29	**0.51**
18	0.04	**0.44**	0.35	0.17	0.32	**0.50**	0.14	0.04	**0.44**	0.36	0.10	0.09	0.06	0.20	**0.40**	0.34
19	0.07	0.39	**0.41**	0.13	0.38	**0.43**	0.13	0.07	**0.47**	0.33	0.04	0.16	0.08	0.26	**0.35**	0.31
20	0.04	**0.43**	0.41	0.11	**0.44**	0.36	0.12	0.08	**0.61**	0.29	0.06	0.03	0.13	0.30	**0.36**	0.21
21	0.11	**0.45**	0.35	0.09	**0.48**	0.38	0.11	0.02	**0.64**	0.25	0.08	0.03	0.17	0.29	**0.40**	0.13
Latent class and membership probability	Female: Class 1				Female: Class 2				Female: Class 3				Female: Class 4			
	Always-to-Rarely				Rarely-to-Never				Always				Rarely			
	33%				34%				10%				23%			
Age	Never	Rarely	Often	Always	Never	Rarely	Often	Always	Never	Rarely	Often	Always	Never	Rarely	Often	Always
9	0.02	0.17	0.35	**0.46**	0.10	**0.41**	0.27	0.22	0.04	0.23	0.28	**0.46**	0.10	**0.51**	0.25	0.13
10	0.01	0.06	0.41	**0.52**	0.12	**0.36**	0.32	0.21	0.04	0.26	0.18	**0.52**	0.06	**0.55**	0.33	0.07
11	0.00	0.07	0.42	**0.51**	0.11	**0.42**	0.32	0.15	0.04	0.22	0.27	**0.47**	0.09	**0.56**	0.31	0.05
12	0.00	0.15	**0.47**	0.38	0.26	**0.46**	0.21	0.07	0.04	0.18	0.27	**0.51**	0.16	**0.60**	0.21	0.03
13	0.03	0.33	**0.35**	0.29	0.37	**0.45**	0.12	0.05	0.00	0.18	0.25	**0.56**	0.06	**0.72**	0.19	0.03
14	0.06	**0.46**	0.27	0.21	**0.46**	0.39	0.06	0.08	0.00	0.24	0.18	**0.58**	0.12	**0.60**	0.17	0.11
15	0.29	**0.38**	0.24	0.09	**0.69**	0.22	0.03	0.06	0.04	0.11	0.31	**0.53**	0.21	**0.54**	0.15	0.10
16	0.18	**0.53**	0.22	0.07	**0.65**	0.25	0.03	0.07	0.00	0.05	0.30	**0.65**	0.15	**0.62**	0.12	0.12
17	0.25	**0.49**	0.18	0.08	**0.70**	0.22	0.03	0.04	0.00	0.18	0.30	**0.52**	0.18	**0.60**	0.10	0.12
18	0.28	**0.46**	0.22	0.04	**0.70**	0.22	0.06	0.02	0.10	0.15	0.32	**0.43**	0.20	**0.56**	0.17	0.08
19	0.32	**0.48**	0.13	0.08	**0.73**	0.22	0.05	0.01	0.16	0.29	**0.37**	0.18	0.21	**0.59**	0.13	0.07
20	0.40	**0.48**	0.10	0.02	**0.74**	0.19	0.06	0.01	0.04	**0.39**	0.35	0.21	0.27	**0.56**	0.12	0.04
21	0.36	**0.39**	0.22	0.03	**0.68**	0.24	0.08	0.01	0.12	0.28	**0.40**	0.20	0.34	**0.48**	0.15	0.03

Bold numbers indicate the highest probability among the four responses in the specific age of each class

Regarding the four latent classes of ESPE for females, Class 1 was labeled as “Always-to-Rarely” and accounted for 33% of females. Females in this class had a high probability of always engaging in ESPE from the age of 9 to 11 years, and an increasing probability of rarely engaging in ESPE from the age of 14 years. Class 2 (34%) was labeled as “Rarely-to-Never” and included female participants who had a high probability of rarely engaging in ESPE at the beginning of the study and a high probability of never engaging in ESPE from the age of 14 years onwards. Class 3 was the smallest class (10%) and labeled as “Always,” as they had the highest probability of always engaging in ESPE over time. However, although the pattern of the “Always” class for females was similar to that for males, the proportion of females was much smaller (10% of females vs. 27% of males). Class 4 was labeled as “Rarely” and included females who had the highest probability of rarely engaging in ESPE over time. The average probability of correctly classifying female participants into each latent class was 0.82, 0.85, 0.89, and 0.82 for classes 1, 2, 3, and 4, respectively. The average level of ESPE by sex and age for every trajectory group is presented in Appendix 2.

### Factors associated with trajectories of ESPE

Table 4 shows the results of multinomial logistic regressions for males and females. Similar results were observed in the sensitivity analysis with complete data based on a multiple imputation strategy (Additional files 1). We use the “Rarely-to-Never” class, the lowest level of ESPE, as the reference group, because we can investigate the factors which can promote participants’ ESPE. The results indicated that males who had higher levels of body dissatisfaction were significantly less likely to be categorized in the “Often-to-Rarely,” “Always-to-Never,” and “Always” classes. Furthermore, males who had higher stress were significantly less likely to be categorized in the “Always-to-Never” and “Always” classes. In addition, males who reported more frequent screen behavior had a lower probability of being categorized in the “Often-to-Rarely” class, but a higher probability of being categorized in the “Always-to-Never” class. Regarding parental factors, males with regularly exercising parents had higher probabilities of being categorized in the “Always-to-Never” class and the “Always” class than did their counterparts.

**Table 4:** Results of the multinomial logit model examining factors related to ESPE trajectories

Males (*n* = 874)	1. Often-rarely / 2. Rarely-Never	3. Always-Never / 2. Rarely-Never	4. Always / 2. Rarely-Never
Factors	OR (95% C. I.)	OR (95% C. I.)	OR (95% C. I.)
*Individual factors*			
Body Mass Index	1.047 (0.993 – 1.105)	0.967 (0.909 – 1.028)	1.030 (0.972 – 1.091)
Body dissatisfaction	0.913 (0.842 – 0.989 )*	0.894 (0.820 – 0.974)*	0.844 (0.777 – 0.918)*
Stress	0.994 (0.955 – 1.034)	0.952 (0.910 – 0.997)*	0.955 (0.914 – 0.997)*
Screen behavior	0.800 (0.682 – 0.937)*	1.184 (1.001 – 1.402)*	0.909 (0.772 – 1.070)
*Parental Factors*			
Parental exercise			
Regular / unregularly or no exercise	1.459 (0.965 – 2.208)	1.600 (1.011 – 2.532)*	1.583 (1.025 – 2.444)*
Parental screen behavior	1.020 (0.870 – 1.195)	1.123 (0.942 – 1.338 )	1.094 (0.925 – 1.295)
Females (*n* = 827)	1. Always-Rarely / 2. Rarely-Never	3. Always/ 2. Rarely-Never	4. Rarely / 2. Rarely-Never
Factors	OR (95% C. I.)	OR (95% C. I.)	OR (95% C. I.)
*Individual factors*			
Body Mass Index	1.080 (1.012 – 1.152)*	1.214 (1.110 – 1.327)*	1.074 (1.001 – 1.152)*
Body dissatisfaction	0.945 (0.875 – 1.020)	0.846 (0.755 – 0.949)*	0.913 (0.841 – 0.991)*
Stress	0.984 (0.947 – 1.023)	0.999 (0.942 – 1.059)	1.008 (0.967 – 1.050)
Screen behavior	0.717 (0.598 – 0.860)*	0.838 (0.645 – 1.089)	0.729 (0.598 – 0.887)*
*Parental Factors*			
Parental exercise			
Regular / unregularly or no exercise	1.272 (0.888 – 1.822)	1.150 (0.668 – 1.980)	1.184 (0.804 – 1.745)
Parental screen behavior	1.033 (0.897 – 1.191)	1.190 (0.970 – 1.459)	0.998 (0.856 – 1.163)

The multinomial logistic model used “Rarely-Never” class as a reference group. Parental highest education, family monthly income, and parental marital status were controlled in the model

*ESPE* extracurricular sport participation and exercise

*p < .05, *OR* Odds Ratio, *C.I.* Confidence Interval

For females, again using the “Rarely-to-Never” class as the reference group, those with a higher BMI level had a higher probability of being categorized in the “Always-to-Rarely,” “Always,” and “Rarely” classes (Table 4). Females who were more dissatisfied with their body were less likely to be categorized in the “Always” and “Rarely” classes. Females who reported more frequent screen behavior were also significantly less likely to be categorized in the “Always-to-Rarely” and “Rarely” classes. No parental behavioral factor was found to be associated with trajectories of ESPE for females.

## Discussion

This study has several key findings. First, four trajectories of ESPE from childhood to emerging adulthood (age of 9 to 21 years) for males and females were identified. For males, these trajectories were Rarely-to-Never, Often-to-Rarely, Always-to-Never, and Always. For females, these trajectories were Rarely-to-Never, Rarely, Always-to-Rarely, and Always. The finding was similar to the results of other studies that focused on the unorganized physical exercise [58] and organized sport participants [59] in that they also found a group of males and females who kept active or who regularly exercised during the follow-up time. In this study, more than a quarter of males frequently exercised over 13 years (“Always” in males), whereas only a tenth of females maintained exercise habits from adolescence to young adulthood (“Always” in females). Despite these observed sex differences, the result indicated that extracurricular exercise habits can be developed and sustained in this age group.

Second, the result indicated that the initial age of decline in regular ESPE was earlier for females (age 11) than for males (age 14) when comparing trajectories of ESPE with similar patterns across sexes (“Always-to-Never” for males and “Always-to-Rarely” for females). Despite previous studies [23–25, 60] consistently showing different patterns of physical activity for males and females, most of them did not examine sex differences in the timing of the decline. Among those that did examine such differences [17, 61], Nelson et al. [61] showed an earlier decrease in physical activity for girls than for boys (declining from early to mid-adolescence versus declining from mid- to late adolescence). However, no specific age of decline was specified. Our study extends previous findings by revealing that females might decrease ESPE at the age of 11 to 13. Therefore, it is essential to develop effective health promotion strategies to encourage ESPE and help young people develop regular exercise habits, especially for females.

Third, some similarities in factors related to ESPE for both sexes were identified. We found that body dissatisfaction was significantly associated with low frequencies of ESPE (i.e., Rarely-to-Never in males and females). In a Chinese society that emphasizes the face-conscious (“mianzi” in Chinese) nature of social life [62], adolescents in our sample who had high body dissatisfaction may be unwilling to exercise because they feel embarrassed when doing so [36]. Screen behavior was the other factor that was associated with low ESPE in both sexes. Extending previous findings on the negative association between screen behavior and physical activity (e.g., Sisson et al., [63]), we demonstrated that screen behavior in childhood could have lasting effects on the initiation and maintenance of ESPE. Therefore, it is vital for both sexes to reduce screen behavior in the early stages of life to increase the likelihood of exercise in the future.

Fourth, we observed sex differences in factors related to ESPE. For individual factors, stress was only associated with trajectories of ESPE for males, and BMI was only associated with trajectories of ESPE for females. Specifically, males with a higher level of stress had a higher chance of developing chronically low frequencies of ESPE (“Rarely-to-Never” compared with “Always-to-Never” and “Always”). Despite the surprising and counterintuitive nature of the observed direction of the association between stress and ESPE, it is likely that males who belonged to the “Rarely-to-Never” class were those who initially had a higher level of stress. Given that our study cannot infer causality, future research should further investigate causal relationships between stress and exercise among males. In addition, the null association between stress and ESPE among girls may be that, unlike males who are more likely to use exercise as a coping strategy for stress, females tend to seek social support or use tension-reduction strategies [41].

We also found that BMI was only associated with trajectories of ESPE for females. The observed sex differences may be due to the fact that females are more concerned about their weight and are more likely to use exercise to control it than are males [64, 65]. Prior studies have also found that the association between BMI and exercise was more apparent among females than males [35, 39]. The nature of the observed association between BMI and ESPE trajectories for females, however, was inconsistent with previous research. Specifically, although other studies have indicated that females with higher BMI were less likely to exercise [35, 39], we found that females with higher BMI were more likely to initiate and maintain ESPE. Factors that may have contributed to these discrepancies include differences in the study design, sample characteristics, analytical strategy, and exercise measurement. Because there are currently no other studies exploring the association between BMI and ESPE trajectories, further research is needed to clarify these relationships.

For parental factors, parental exercise was only associated with ESPE trajectories for males, such that males with parents who regularly exercise were more likely to engage in high-frequency ESPE (i.e., “Always-to-Never” and “Always” compared with “Rarely-to-Never”). Our findings are consistent with other studies which revealed that the effects of parental exercise behavior were only significant among boys (Moor et al., [44]; Cleland et al., [43]). One possible mechanism to explain how parents’ exercise behavior influences ESPE for males is “role modeling” as indicated by social-learning theory [33, 66]. Specifically, when offspring observed that their parents exercise frequently, which entails positive values of sport and exercise, they may embrace these values and exert similar behaviors. The observed exercise behavior from parents may also serve as a prompt for children’s participation in ESPE. Additionally, as research has found that parents who are physically active are more likely to support their children in physical activity [29], it is possible that parental exercise behavior influences males’ ESPE indirectly via parental support in ESPE. Lastly, genetic predisposition to physical activity may help explain the observed association. Research [44, 67] has shown that there is a certain amount of heritability for sports participation. Therefore, offspring who have more ESPE may do so partly because of the genetic factors inherited from their active parents.

Despite the fact that we did not find a significant association between parental exercise behavior and ESPE among females, our results do not mean that parents do not have any influence on females’ ESPE. Research has suggested that having a parent to participate in ESPE with, rather than observing their behavior, may be more important for promoting ESPE among females [43]. It is also likely that female participation in ESPE is affected more by parental attitudes to exercise behavior than by parental behavior [68]. As we did not measure other ESPE-related parental influences, more research is needed to further understand whether and how parental factors influence their offspring’s ESPE differently for males and females.

Our study has some notable strengths. First, the use of longitudinal data allowed us to delineate the sex-specific development of ESPE across multiple stages of life. Second, by applying a sophisticated data analysis, we could capture distinct developmental patterns and factors associated with ESPE. Specifically, we identified important sex differences in both the timing of the decline and the factors associated with the trajectories of ESPE for males and females. Therefore, our findings not only provide important information regarding the identification of potential high-risk groups, but also help develop sex-specific strategies for intervention. For example, the design of a physical activity enhancing program tailored to females in the “Rarely-to-Never” group by focusing on reducing the effect of body dissatisfaction and increasing self-monitored behavior might significantly increase women’s physical activity participation [69].

Several limitations of the study also need to be addressed. For instance, exercise was measured using a single retrospective self-reported question that may be subject to recall bias. Second, because our sample was drawn from northern Taiwan, the generalizability of our findings may be limited. Thus, caution should be exercised when applying the current results to other populations. Third, the CABLE project did not measure the duration of ESPE; therefore, the trajectories can only represent the frequencies of exercise. However, the diversities of duration of ESPE, e.g. 15 min or an hour, may contribute differently to the recommendation of physical activity. Thus, future study should estimate the duration and frequency of ESPE simultaneously to increase the precision. Fourth, the level of ESPE might be overestimated if some respondents reported the frequency of physical activity. Last, ESPE and parental exercise were measured by one question that did not capture the exercise quantity. Future studies that measure ESPE and parental exercise using other validated assessments are needed to confirm our results.

## Conclusions

This study identified four trajectories of ESPE for males and females, respectively. The results enhance the existing knowledge by revealing the initial ages of decline in ESPE and demonstrating the sex-specific individual and parental factors which contribute to the distinct trajectories of ESPE. Our results could also benefit the future development of the ESPE promotion programs because we revealed factors that help maintain a high level of ESPE. Additionally, because factors associated with ESPE are different for males and females, interventions should be tailored accordingly to increase their effectiveness.

### Supplementary Information

**Additional file 1.** Results of multinomial logit model examining preadolescent factors related to exercise trajectories using complete data created by multiple imputation analysis.

## Figures and Tables

**Fig. 2:**
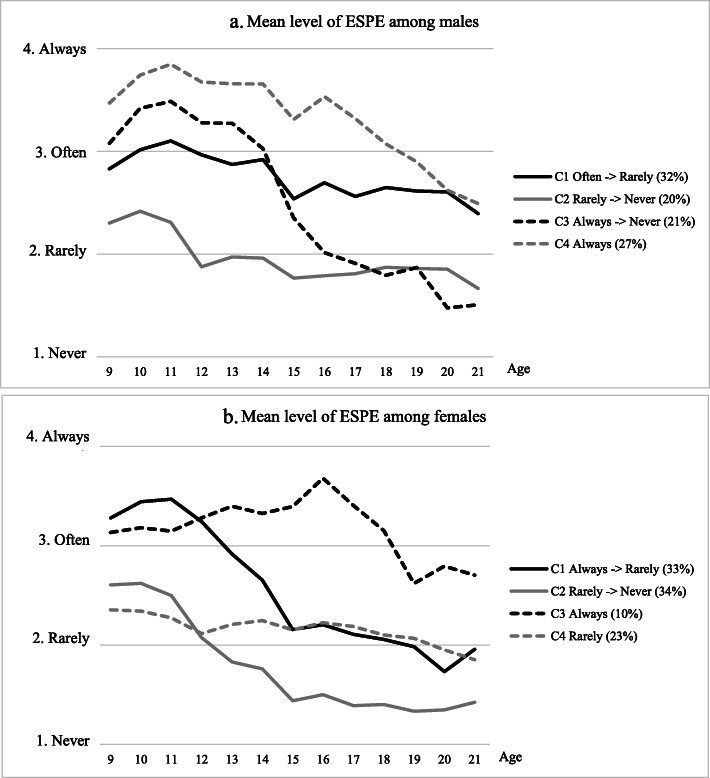
The average level of ESPE by sex and age for every trajectory group. ESPE: extracurricular sport participation and exercise

## Data Availability

The data used in the present study can be made available on request to the correspondence authors.

## Appendix 1

The calculation of inter-method reliability of participants’ extracurricular sport participation and exercise.

To understand the reliability of our self-reported measure of ESPE, we conducted a correlation analysis between the one-item ESPE measure with another three-item measure of physical activity (PA) assessed in the 12th wave. The three-item measure was adopted from the questions used in the chronic disease risk factor surveillance (STEPS) [70] as below:

1. On average, how many times you do exercise in 1week? (The exercise has to be continued for more than 10 min) _____ time(s)
2. On average, how many minutes do you spend on the exercise every time? _____ minute(s)
3. When you do exercise, will you feel shortness of breath? (1) nothing changed (2) small increases in breathing or heart rate (3) panting (4) out of breath

We used these three questions to define if a person meets the WHO’s PA recommendation that “Adults aged 18–64 years should do at least 150 minutes of moderate-intensity aerobic physical activity throughout the week” because our participants were 20 years old in the 12th wave of the survey. We defined Q3 > =2 as moderate-intensity PA. Q1*Q2 was used to calculate the time of PA. IF Q3 > =2 and time of PA > =150 then the participants were defined as a person who met the WHO’s recommendation.

The results of the correlation analysis are presented in the table below. We observed a significant gradient association between the two measurements (Cochran-Armitage Trend Test: T = 21.1503, *p* < 0.001). Specifically, we found that more than 60% of the participants who reported “often” and “always” to the one item question of this study met the WHO’s recommendation. In addition, about one fifth of the participants who reported “rarely” met the WHO’s recommendation. However, only 1.1% of the participants who reported “never” met the WHO’s recommendation.

**Table:**

Met WHO_PA recommendation (%)	wave_12 ESPE			
	1.Never	2.Rarely	3.Often	4.Always
Yes	1.13	21.62	64.34	69.83
No	98.87	78.38	35.66	30.17

## Abbreviations

ESPE: Extracurricular sport participation and exercise
BMI: Body mass index
