# Supplementary material for: Sex differences in the trajectories of and factors related to extracurricular sport participation and exercise: a cohort study spanning 13 years
Source: BMC Public Health. 2020 Nov 2;20:1639. doi: 10.1186/s12889-020-09745-8 (PMC7607706; doi:10.1186/s12889-020-09745-8)
Supplement: Supplementary file 1 — Additional file 1. Results of multinomial logit model examining preadolescent factors related to exercise trajectories using complete data created by multiple imputation analysis. [file 12889_2020_9745_MOESM1_ESM.docx]

Additional file 1 Results of multinomial logit model examining preadolescent factors related to ESPE trajectories using complete data created by multiple imputation analysis

| **Males** (n=1075) | 1. Often-rarely /  2. Rarely-Never | | | | |  |  | 3. Always-Never /  2. Rarely-Never | | | | | |  | 4. Always /  2. Rarely-Never | | | | | |  |
| --- | --- | --- | --- | --- | --- | --- | --- | --- | --- | --- | --- | --- | --- | --- | --- | --- | --- | --- | --- | --- | --- |
| Factors | OR |  | 95% C. I. | | |  |  | OR |  | 95% C. I. | | |  |  | OR |  | 95% C. I. | | |  |  |
| *Individual factors* |  |  |  |  |  |  |  |  |  |  |  |  |  |  |  |  |  |  |  |  |  |
| Body Mass Index | 1.043 | ( | 0.992 | - | 1.097 | ) |  | 0.979 | ( | 0.925 | - | 1.037 | ) |  | 1.039 | ( | 0.984 | - | 1.096 | ) |  |
| Body dissatisfaction | 0.900 | **(** | 0.834 | **-** | 0.972 | **)** | ***** | 0.885 | **(** | 0.814 | **-** | 0.962 | **)** | ***** | 0.832 | **(** | 0.766 | **-** | 0.903 | **)** | ***** |
| Stress | 0.993 | ( | 0.956 | - | 1.032 | ) |  | 0.953 | **(** | 0.913 | **-** | 0.996 | **)** | * | 0.961 | **(** | 0.921 | **-** | 1.003 | **)** |  |
| Screen behavior | 0.841 | **(** | 0.733 | **-** | 0.965 | **)** | * | 1.184 | **(** | 1.021 | **-** | 1.373 | **)** | * | 0.907 | ( | 0.786 | - | 1.046 | ) |  |
| *Parental Factors* |  |  |  |  |  |  |  |  |  |  |  |  |  |  |  |  |  |  |  |  |  |
| Parental exercise |  |  |  |  |  |  |  |  |  |  |  |  |  |  |  |  |  |  |  |  |  |
| Regular / unregularly or no exercise | 1.774 | **(** | 1.200 | **-** | 2.622 | **)** | * | 1.863 | **(** | 1.207 | **-** | 2.875 | **)** | ***** | 1.847 | **(** | 1.227 | **-** | 2.781 | **)** | ***** |
| Parental screen behavior | 0.946 | ( | 0.824 | - | 1.086 | ) |  | 1.028 | ( | 0.882 | - | 1.198 | ) |  | 1.064 | ( | 0.920 | - | 1.230 | ) |  |
| **Females** (n=997) | 1. Always-Rarely / 2. Rarely-Never | | | | |  |  | 3. Always/ 2. Rarely-Never | | | | | |  | 4. Rarely/ 2. Rarely-Never | | | | | |  |
| Factors | OR |  | 95% C. I. | | |  |  | OR |  | 95% C. I. | | |  |  | OR |  | 95% C. I. | | |  |  |
| *Individual factors* |  |  |  |  |  |  |  |  |  |  |  |  |  |  |  |  |  |  |  |  |  |
| Body Mass Index | 1.077 | **(** | 1.015 | **-** | 1.141 | **)** | ***** | 1.179 | **(** | 1.089 | **-** | 1.277 | **)** | ***** | 1.081 | **(** | 1.014 | **-** | 1.152 | **)** | ***** |
| Body dissatisfaction | 0.946 | ( | 0.877 | - | 1.021 | ) |  | 0.837 | **(** | 0.751 | **-** | 0.933 | **)** | ***** | 0.897 | **(** | 0.827 | **-** | 0.972 | **)** | ***** |
| Stress | 0.988 | ( | 0.952 | - | 1.024 | ) |  | 1.007 | ( | 0.952 | - | 1.064 | ) |  | 1.005 | ( | 0.964 | - | 1.047 | ) |  |
| Screen behavior | 0.714 | **(** | 0.608 | **-** | .838 | **)** | ***** | 0.937 | ( | 0.751 | - | 1.168 | ) |  | 0.830 | **(** | 0.698 | **-** | 0.986 | **)** | ***** |
| *Parental Factors* |  |  |  |  |  |  |  |  |  |  |  |  |  |  |  |  |  |  |  |  |  |
| Parental exercise |  |  |  |  |  |  |  |  |  |  |  |  |  |  |  |  |  |  |  |  |  |
| Regular / unregularly or no exercise | 1.173 | ( | 0.840 | - | 1.637 | ) |  | 1.143 | ( | 0.688 | - | 1.900 | ) |  | 1.073 | ( | 0.746 | - | 1.542 | ) |  |
| Parental screen behavior | 1.013 | ( | 0.894 | - | 1.149 | ) |  | 1.212 | **(** | 1.012 | **-** | 1.450 | **)** | ***** | 1.006 | ( | 0.876 | - | 1.155 | ) |  |

The multinomial logistic model used “Rarely-Never” class as a reference group. Parental highest education, family monthly income, and parental marital status were controlled in the model.

ESPE: extracurricular sport participation and exercise

*: p < .05, OR: Odds Ration, C.I.: Confidence Interval
